# What affects unplanned hospital admissions in older adults according to primary healthcare professionals? A focus group study

**DOI:** 10.1080/13814788.2026.2650928

**Published:** 2026-05-07

**Authors:** Jet H. Klunder, Eline C. M. Kooijmans, Karlijn J. Joling, Otto R. Maarsingh, Hein P. J. van Hout

**Affiliations:** a Department of General Practice, Amsterdam UMC, Vrije Universiteit Amsterdam, Amsterdam, Netherlands; b Aging and Later Life, Amsterdam Public Health, Amsterdam, Netherlands; c Department of Medicine for Older People, Amsterdam UMC, Vrije Universiteit Amsterdam, Amsterdam, Netherlands

**Keywords:** Older adults, hospital admission, primary care professionals, qualitative research

## Abstract

**Background:**

Unplanned hospital admissions are distressing for older persons and are associated with a high risk of adverse outcomes and a burden on health systems. Understanding the risk and protective factors for unplanned admissions can help to design new preventive interventions in primary care.

**Objectives:**

To explore primary healthcare professionals’ experiences on factors affecting unplanned hospital admissions in community-dwelling older adults and identify opportunities for preventive interventions.

**Methods:**

We performed a focus group study with a purposive sample of Dutch primary healthcare professionals. Four focus groups were conducted with a total of 22 primary healthcare professionals comprising 10 general practitioners (GPs) and 12 other primary healthcare professionals. All focus groups were recorded, transcribed, and thematically analysed.

**Results:**

Factors affecting unplanned admissions were grouped into characteristics of the patient, the healthcare professional, and healthcare organisation. Patient-related risk factors included the presence of chronic conditions, health-seeking behaviour, the presence and capacity of an informal caregiver, and cultural expectations of healthcare. Continuity of care, advance care planning, and professional experience as a GP were identified as mitigating professional-related factors for unplanned admissions. Organisational factors that potentially contributed to unplanned admissions were poor informational continuity, suboptimal care coordination, and lack of alternatives to hospitalisation.

**Conclusion:**

Unplanned hospital admissions in older adults were perceived to be influenced by patient, healthcare professional, and healthcare organisation-related factors. Strategies such as ensuring broad access to patients’ clinical information and treatment wishes, improving personal continuity of care, and structural provision of advance care may contribute to reduce unplanned admissions.

## Introduction

The ageing population presents challenges to healthcare systems worldwide, with unplanned hospitalisations among older adults being a growing concern. Consequences include potential adverse effects on patient’s quality of life, overcrowded emergency departments (EDs), and increased healthcare costs [1]. Simultaneously with an increased demand for healthcare, there is a growing shortage of staff across the healthcare sector [2]. Therefore, a shift in focus from reactive and fragmented care to proactive and coherent care has been advocated [3]. Identification of older adults at high risk of unplanned admissions could be a valuable first step towards preventing unplanned admissions through targeted interventions [3].

Primary care providers are often the first point of contact for community-dwelling older adults, making them well-positioned to identify frail individuals and target preventive interventions. In the Netherlands, general practitioners (GPs) handle about 95% of health complaints and offer chronic disease management of highly frequent disorders [4]. As such, they are instrumental in ensuring patients to receive appropriate and timely care. In the care for older adults, GPs increasingly collaborate in a multidisciplinary network with general practice-based nurses and community-based healthcare professionals.

Numerous observational studies have identified risk factors associated with unplanned admissions in older adults, such as comorbidities, polypharmacy, and previous admissions [5–7]. However, the nuances and contextual factors that influence primary healthcare professionals’ decision-making processes and care management strategies are generally not captured by quantitative data alone. Exploring experiences of professionals may help to better understand unplanned hospital admissions. This could offer valuable insights into both risk factors and potential targets for preventive interventions.

The aim of this study was to explore risk factors for unplanned hospital admissions in community-dwelling older adults as perceived by primary healthcare professionals and identify opportunities for preventive interventions.

## Methods

### Setting

This study was conducted with healthcare professionals working in Dutch primary care. GPs are the primary point of contact for medical care, Dutch inhabitants are registered at a single general practice, and GP care is covered by mandatory health insurance. The Netherlands employs a gatekeeping system that requires a GP referral to access specialty care [8]. Out-of-hours, patients can contact GP cooperatives for urgent issues. In emergencies, patients can also directly present themselves to the ED or call the national emergency number.

Nine out of ten people aged ≥65 years visit their GP at least once a year, with an average of eight consultations annually [9]. In addition to GPs, other primary healthcare professionals, such as general practice-based nurses, district nurses, and case managers with expertise in dementia provide comprehensive care for older adults. General practice-based nurses, supervised by GPs, offer health education and support in managing chronic conditions. District nurses provide personal health support and nursing care in the patients’ homes. In cases of dementia, case-managers specialised in dementia may be involved to coordinate care and support for patients and their caregivers.

### Study design and sampling

A qualitative study involving focus group discussions was performed to explore risk and protective factors of unplanned hospital admissions in community-dwelling older adults and to identify opportunities for preventive interventions. We recruited primary healthcare professionals, specifically GPs and nurses working in general practice and community care. Nurses working in an Acute Geriatric Community Care Hospital (AGCH) were also invited. The AGCH provides acute hospital-level care for older adults with acute medical conditions within a skilled nursing facility [10]. While non-GP professionals are often not directly involved in the hospitalisation process, their regular patient interactions play an important role in signalling deterioration and urgent care needs that may result in unplanned admissions. We will refer to these non-GP professionals as *other healthcare professionals* (OHCPs).

To reduce potential hierarchy and encourage free and open discussions, focus groups were conducted separately with the GPs and OHCPs [11,12]. Our study was reported in accordance with the Standards for Reporting Qualitative Research Checklist [13].

### Recruitment and data collection

A purposive sample was composed by approaching primary care professionals from the researchers’ professional networks and through a call in the newsletter of the Academic Network of General Practice Amsterdam UMC. Participants received written information through email. A small reimbursement was offered for time compensation. All participants gave informed consent by email and completed a short questionnaire before the focus group.

Four focus groups were conducted between January 2021 and April 2021: two with GPs and two with OHCPs. Due to the COVID-19 pandemic, the focus groups were held online, we aimed for a maximum of six participants per session [14].

Participants were asked to recall recent cases involving older patients’ unplanned hospitalisations. During the focus group, participants presented their cases, followed by a guided discussion using a flexible topic guide. This allowed for exploration of relevant and new unanticipated issues (Supplementary File 1). The focus groups were moderated by a skilled and independent non-practicing, male GP, while an assistant moderator (JHK) took notes and summarised the discussion. Each focus group lasted ∼90 min. Following each focus group, a detailed summary was reviewed by JHK, HPJvH, and the moderator to refine the topic guide, following the principles of research as an iterative and reflexive process [15].

### Analysis

All focus groups were video-recorded and transcribed verbatim and anonymously. Thematic analysis with an inductive approach was used to identify and report themes within the data [16]. All transcripts were read in detail and independently coded by two researchers (JHK, ECMK) using MAXQDA 2020, followed by comparing the codes. Disagreements were resolved through discussion. In case of persistent disagreement, a third researcher (HPJvH) was consulted. Preliminary themes and subthemes were discussed within the research team resulting in an analytical framework. The final step was producing a comprehensive report of relevant segments for each (sub)theme and selecting the most compelling ones.

### Reflexivity

At the time of the focus groups, JHK (female GP trainee/PhD student) and ECMK (female MD/PhD student) had limited prior experience in qualitative research but had formal training in qualitative research. To enhance methodological rigour, coding was initially discussed with an experienced qualitative researcher (HPJvH).

Both JHK and ECMK brought clinical experience from hospital and primary care settings that likely shaped their analytical lens. JHK had worked in geriatric and emergency departments, which motivated this research, though this hospital-based perspective predominantly exposed her to patients after admission had already occurred. ECMK’s experience in nursing homes and rehabilitation centres similarly provided insight into post-admission trajectories. While JHK’s primary care experience provided some counterbalance, these clinical backgrounds may have led to greater emphasis on codes aligned with personal professional experiences.

The multidisciplinary research team, including senior researchers with backgrounds in primary care (HPJvH), general practice (ORM, also practicing GP), and medicine for older people (KJJ) helped balance these perspectives. Team members without clinical backgrounds (HPJvH, KJJ) challenged medical assumptions and ensured interpretations remained accessible to non-clinical audiences. Regular team discussions throughout the analysis process helped identify and examine potential biases arising from these varied positions.

## Results

### Demographic characteristics

Twenty-two professionals participated: ten GPs and twelve OHCPs (Tables 1 and 2). One GP was included through the newsletter, the other participants through the professional networks. Participants were based in the Amsterdam area (*n* = 11), south (*n* = 6), central (*n* = 4), and north (*n* = 1) of the Netherlands. Time constraints were the main reason for non-participation.

**Table 1.** Composition of focus groups including the professions of the different healthcare professionals.

**Focus group 1 (*n* = 5)**	**Focus group 2 (*n* = 4)**
General practitioner (*n* = 5)	General practice-based nurse
	Nurse specialist in district nursing
	Case-manager dementia (*n* = 2)
**Focus group 3 (*n* = 8)**	**Focus group 4 (*n* = 5)**
General practice-based nurse	General practitioner (*n* = 5)
District nurse (*n* = 2)	
Nurse specialist in district nursing	
Case-manager dementia (*n* = 2)	
Nurse (practitioner) in AGCH (*n* = 2)	

AGCH: Acute Geriatric Community Care Hospital.

**Table 2.** Characteristics of focus group participants.

	GPs (*n* = 10)	OHCPs (*n* = 12)
**Age, range (years)**	34–60	32–57
**Age (years), *n***		
31–40	2	1
41–50	1	4
51–60	7	7
**Sex, *n***		
Female	9	11
Male	1	1
**Working experience, range (years)**	3–24^a^	2–31^b^
**Years of working experience, *n***		
2–10	3	7
11–20	1	2
≥20	6	3

GP: general practitioner; OHCP: other primary healthcare professional.

^a^ As a GP.

^b^ In their current profession.

### Main themes

**Figure 1.**
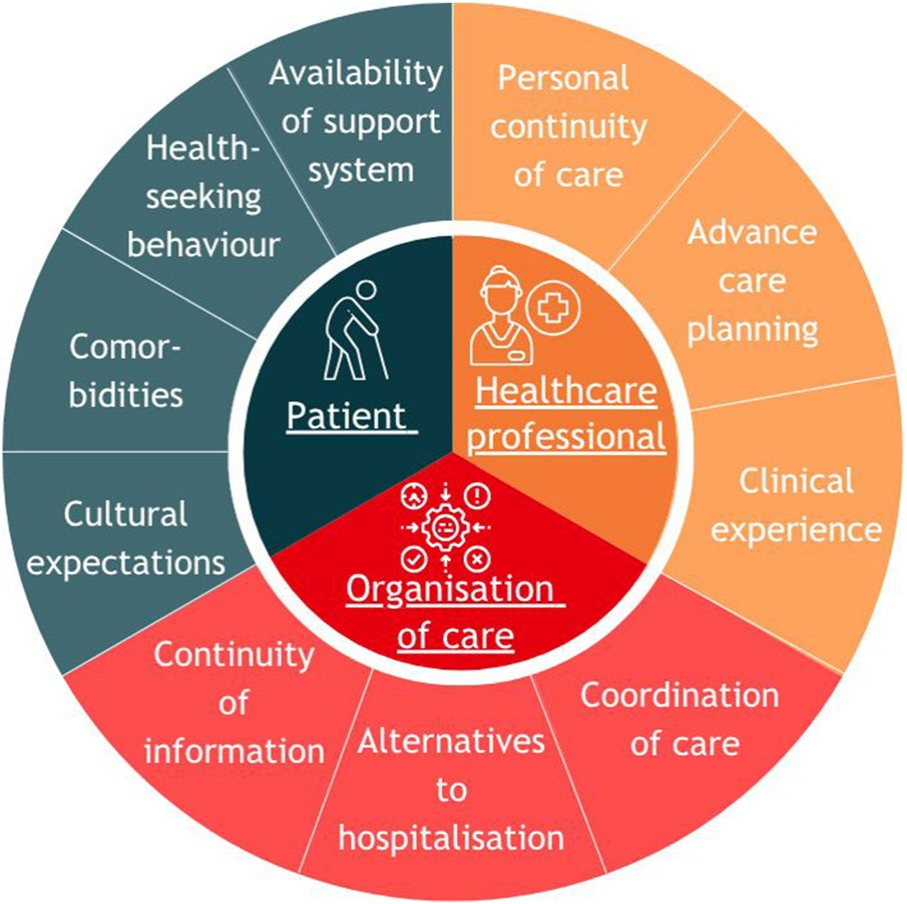
Visual presentation of the main themes and subthemes.

After analysis, we grouped themes into patient, healthcare professional, and healthcare organisation characteristics (Figure 1, Supplementary File 2).

#### Patient

##### Comorbidities

Patients often suffered from multiple chronic conditions, frequently accompanied with polypharmacy. Other psychosomatic factors that came up were cognitive decline and dementia, anxiety, depression, and alcohol abuse.

*Moderator: If we summarise the types of patients (at risk of hospitalisation); dementia of course, […] falls are a major issue. Heart failure, Chronic Obstructive Pulmonary Disease (COPD), and diabetes have been mentioned often […] OHCP1: I would say polypharmacy, persons taking a lot of medication are at risk. And I have many patients that drink a lot of alcohol. OHCP2: Yes, and multimorbidity in particular. There is often a lot going on at the same time in terms of characteristics, including somatic issues, but also in the context […] like socially, functionally, many things.*

##### Availability and capacity of the support system

The availability and capacity of a support system affected participants’ decision-making in acute care situations.

*Good informal care and a good support system is very important. If someone lives alone and has no support at all, to me that is an alarm symptom, because there is no back-up. If things go wrong, there is nobody to call. And there is also nobody who signals cognitive decline. (OHCP2)*

The caregiver was considered an important spokesperson for the patient when decisions had to be made. The caregiver’s interest whether or not to refer the patient to the ED (*sometimes against the patient’s wishes,* GP8) also affected the GP’s decision-making. For instance, in case of overburdening.

*That happens often; informal caregivers who are overburdened, […] often severely, end up not being able to cope with the stress of taking care anymore. So, they put their foot down and say my mother should be hospitalised. (OHCP11)*

Conversely, the informal caregiver, often children, could also influence the patient’s wishes.

*The older generation themselves, in their eighties or nineties, often express the desire to not be hospitalised anymore. […] However, their children may say ‘Come one Dad, you have to move on’ showing reluctance to let go. In such cases the client often goes along with the child’s preference. At times, I question whether this is truly what the patient wants and if it’s beneficial to continue with their treatment. (OHCP2)*

##### Health-seeking behaviour

In many cases, ‘care avoiders’ were presented as typical patients at higher risk of unplanned hospitalisations. These patients usually withhold any care, often suffer from addictions (particularly alcohol) or lack a support network. Participants struggled with how to provide appropriate care:

*If I undertook two home visits (and the patient still does not take my advice), then it kind of stops after that. (GP3) Yes, exactly, with me too. (GP2)*

Professionals reflected on how to balance between respecting the patient’s autonomy in (not) seeking healthcare (timely) with accepting the risks involved. They mentioned older people often know too little about their illness and when to raise the alarm. Consequently, they carry on with their symptoms for too long, sometimes making hospitalisation unavoidable.

*I know many patients with COPD, often older men who smoke, that usually alert too late with their complaints. They think ‘it will pass’ or ‘tomorrow will be better, I just need some sleep’, but often it’s too late. […] How do you reach these people and educate them, that they get help before the exacerbation? (OHCP1)*

##### Cultural expectations of healthcare

Explaining to people from diverse cultural backgrounds that hospitalisation is not always the best solution was found to be challenging. Participants experienced these patients often have different treatment expectations and preferences influenced by their cultural heritage.

*There’s a large population with distinct cultural backgrounds who usually want a referral to emergency care, because in their view you cannot withhold treatment. […] And sometimes I share my opinion (on averting hospitalisation), but I do refer them to the ED. And yes, the decision, whether this was justified or not, then has to be discussed in the hospital as well. But you cannot say, I won’t refer you. (GP8)*

#### Healthcare professional

##### Personal continuity of care

The importance of cultivating a strong relationship with patients was expressed by the majority of participants. District nurses and case-managers stressed the need to get to know the patient, including their mental and physical health, behaviour and mood, social relations, and home environment. They noted that knowing your patients’ interests and motivations builds mutual trust and facilitates decision-making. GPs indicated this cannot be overcome by *a perfect patient record* (GP6) and reported the key to maintaining a good doctor-patient relationship is personal continuity.

GP6 described a case where discontinuity of care was the cause for repeated admissions. The patient had already been analysed by a neurologist three times without a clear diagnosis.

*When I saw this clinical picture (i.e. recurrent vertigo with a suspicion of a central cause) for the first time, I wondered if I should presume it was the same as before. I opted not to because the symptoms were very severe. Therefore, the neurologist reviewed him again but couldn’t give a definite diagnosis. […] A week later, an out-of-hours GP referred him to the hospital again with the same picture. So, this patient visits different doctors with an urgent, but unclear clinical issue, leading to repeated behaviour without getting a diagnosis. […] And I believe improved continuity would facilitate direct communication between the GP and the specialist, resulting in a timely care plan, which would lead to a better patient outcome after only one or two admissions. (GP6)*

In this example, the lack of personal continuity also resulted in fragmented information, illustrating the close connection between personal and informational continuity in daily practice.

Two GPs mentioned that because of personal discontinuity, it is sometimes unclear who has the primary responsibility for the patient. Designating a doctor to be responsible for the coordination of the patient’s care and keeping the electronic health record (EHR) up to date were strategies the GPs used to tackle the issues caused by lack of personal continuity.

##### Clinical experience

The case described above also highlights the clinical uncertainty and responsibility that GPs face, particularly in the context of acute care. In order to be more reassured, some GPs decided to consult a medical specialist for further diagnostics, leading to hospitalisation. With years of experience, this uncertainty seemed to decrease.

*And I also notice, after 25 years as a GP, you dare to take more risk. (GP7) [GP9, 10+ years of experience nods] And sometimes you also dare to think it is better for people to stay at home. I think when you’re young, you tend to be more proactive (and avoid risk). Whereas as you get older, you dare to sit back. (GP9)*

##### Advance care planning

Advance care planning (ACP) was considered an important facilitator in decision-making. This included timely discussion of the patient’s treatment wishes and documenting them in the EHR, discussing the options of healthcare provision and the patient’s expectations of these options, but also informing the patient about what they can expect regarding their health and when to ask for medical help. Participants stated that this is preferably discussed in the presence of a relative, as their preferences could sometimes influence the patient’s wishes.

*What sometimes scares me is the social environment. When you’ve had a good ACP discussion with the patient, and you feel everything is on track […] But then things are brought up again, because the family, the neighbour, or fill in the blanks wants something else. (GP7)*

Next to discussing treatment plans, participants believed it is also important to acknowledge the finiteness of life.

*There is always endless treating instead of leaving […] We should ask the patient what does a good death mean to you? And we should discuss that together with the family […] and that the patient’s wishes should be made possible. And sometimes that means we don’t refer, because they would die in hospital, alone in a white bed and that’s a great pity. (GP9)*

However, advance care plans can be subject to change and should therefore be discussed regularly. GPs experienced this as time-consuming, and valued the role of the practice-based nurse who can take a lot of work off.

*Without the practice nurse, it would just be good intentions. They also have ACP discussions or at least raise the subject to patients. I think they are of huge added value. […] They act as a go-between, a confidant. And I think, because of the practice nurse, we really know a lot about our patients. (GP7)*

#### Healthcare organisation

##### Continuity of information

Poor information continuity was considered an important factor affecting unplanned admissions. This included the information flow with the GP cooperatives and between primary and secondary care. During out-of-hours, GPs working at a GP cooperative have access to patient’s EHR through the National Exchange Point (Landelijk Schakelpunt (LSP) in Dutch). However, participants reported it often lacks information or the system is not operable.

When working out-of-hours, GPs appreciated when a patient summary or a note about treatment wishes was available through the LSP. At the same time, they admitted that they themselves do not record this for most of their patients. Time constraints were the main reason.

*And I can immediately take my share of the blame, I don’t document summaries of all these patients. I definitely do it for palliative patients. But for all the frail patients of whom you’re actually not surprised on Monday when they’ve been admitted during the weekend […] I think for less than a quarter a professional summary and treatment plan is available. (GP3)*

##### Coordination of care

Participants reported that poor collaboration and coordination between primary and secondary care often caused unplanned hospitalisations. They described different perspectives (i.e. person-centred in primary care *vs.* disease-oriented in secondary care) as a contributor—particularly regarding readmissions. Participants found that older patients are often discharged too soon without any aftercare being organised. Many professionals were particularly troubled by the poor communication from the hospital which had sometimes led to readmissions. They preferred a ‘warm’ clinical handover (i.e. with personal contact).

*Ideally, I would have undertaken a home visit immediately after discharge. But I had not received a clinical handover from the hospital. I did not even know she was home already. (GP1)*

GPs also indicated that when a patient is seen by a medical specialist on outpatient basis, the GP tends to leave the care entirely to the specialist, losing sight of the patient. They realise this is often a pitfall and mentioned the need for closer collaboration between the GP and the medical specialist.

*The patient has an annual appointment with the cardiologist, during which they often modify the hypertension medication without evaluating the outcome. So, I think there is a lot of poor follow-up resulting in avoidable admissions. (GP5) And that makes you feel like you don’t have an active role. But that’s a false conclusion, that you think ‘this is being monitored by the cardiologist’, but it’s not happening, or not happening sufficiently. (GP4)*

##### Alternatives to hospitalisation

Furthermore, alternatives to hospitalisation, such as home-based nursing, short-term institutional stays, or admission to a nursing home were frequently not available. Participants attributed this to shortage of beds and staff, as well as to legislation, and stressed the need for a more stable solution.

*And it seems that a hospital is probably not the best place in this case, but there are no alternatives these days. The Long-Term Care Act ensures that fewer people get a place in a long-term care facility. And then, when a patient falls, they have to be admitted somewhere temporarily, which is going to be the hospital. But after that, they get discharged home and will probably fall again. (OHCP7)*

District nurses and case managers emphasised the importance of their timely involvement. If it happens too late, they may be unable to connect with patients. To do their job properly building trust with patients is essential.

*If you’re timely involved, for instance when the diagnosis of dementia has just been made, or hasn’t been made yet, you get to know the person behind the dementia. […] If you’re involved at a later stage, the dementia becomes the main concern, and you cannot connect with the client and have to rely on the informal caregivers. And by then, very often, you are already doing damage control and facing crises. (OHCP4)*

## Discussion

This study explored the perceptions of primary healthcare professionals on factors affecting unplanned hospital admissions in community-dwelling older adults and identified potential preventive interventions. Factors related to the patient, the healthcare professional, and the healthcare organisation impacted on decisions to refer patients to hospital. Providing appropriate medical care while respecting the patient’s or relative’s wishes was found to be challenging. Improving continuity and coordination of care was perceived to possibly prevent unplanned hospitalisations. Provision of timely ACP, such as self-education and discussing treatment preferences, could divert emergency admissions. To achieve this, better collaboration within and between primary and secondary healthcare sectors is necessary.

### Strengths and limitations

Our findings show that organisational and communication challenges, in addition to clinical risk factors, play a key role in unplanned admissions. While previous studies have focused mainly on patient-level predictors, our study provides insight into how these factors interact in daily practice and highlights the importance of system-level prevention strategies. Including primary care professionals not directly involved in decision-making provided a broad perspective. Conducting the focus groups online engaged participants from urban and rural regions of the Netherlands, facilitating a comprehensive view of the identified themes.

This study also has limitations. Most participating GPs were experienced and working in permanent practice positions. While younger or locum GPs were included, their perspectives may be less prominent. These clinicians may encounter different contextual pressures, such as more frequent out-of-hours work and reduced continuity of care. The male perspective is also underrepresented, however, the gender distribution of our sample reflects that of the Dutch healthcare workforce [17]. Furthermore, the study focused solely on primary healthcare providers, including the perspectives of patients, their caregivers, and secondary healthcare providers would have enhanced the comprehensiveness of our findings. This consideration is particularly important in the design of preventive interventions, as deeper understanding of patients’ perspectives may lead to better outcomes. However, our study aimed to explore risk factors for unplanned hospitalisations and to identify opportunities for preventive interventions. We focused on primary healthcare providers given their crucial role in both identifying risk factors and preventing unplanned admissions.

### Comparison with existing literature

Previous qualitative studies have described factors affecting GPs’ decisions about hospitalisation, including the support system, information continuity, clinical uncertainty influenced by experience, and alternatives to hospitalisation [17–19]. This study confirms these findings and highlights the importance of interdisciplinary teamwork. Multimorbidity and polypharmacy have been shown to be important predictors of unplanned hospital admissions in older adults [5–7]. This study sheds light on the underlying dynamics of these admissions, suggesting that organisational and coordination issues, rather than clinical complexity alone, often lead to hospitalisation in this population.

Furthermore, a UK study assessed the decision-making process for unplanned hospitalisations among professionals working in social, primary, and emergency care and identified poor information flow as an important determinant [19]. This encompassed both IT aspects and the lack of communication between different healthcare professionals. Similarly, a Dutch study found that emergency physicians often admitted older adults due to a lack of information from primary care [20]. Our findings emphasise the imperative of better information-sharing and access to relevant patient data, including treatment preferences.

Moreover, similar themes emerged from a study with secondary care physicians [20]. These included the impact of external pressures on patient admissions, e.g. social circumstances, the need for clear patient instructions to initiate timely care-seeking, and the importance of early recognition of declining health in primary care. While the participants in our study advocated a more comprehensive patient-centred approach within hospitals, the secondary care physicians emphasised the need to better anticipate exacerbations in primary care. Notably, both studies called for improving alternatives to hospitalisation. The importance of promoting enhanced interdisciplinary collaboration was also underlined by a qualitative study exploring primary care professionals’ perspectives on person-centred care to prevent hospitalisations [21]. Ultimately, an integrated, patient-centred approach that ensures continuity and coordination of care across social, primary, and secondary care is needed to effectively prevent unplanned hospital admissions in older adults.

### Implications for research and practice

Our study confirms the critical role of continuity of care, patient education, and care coordination in potentially preventing unplanned hospitalisations in older adults. It highlights the need to understand each patient’s needs and treatment preferences, as part of a person-centred care approach. Since greater continuity of care is associated with lower healthcare use and increased physician productivity [22,23], primary care providers should prioritise strategies to improve personal continuity, such as working in small teams of GPs, assigning a GP or practice-based nurse to patients at high risk of hospitalisation and encouraging patients to see their usual GP [24]. Moreover, educating patients and their caregivers about their conditions and when to seek help could enable proactive management and prevent hospitalisation. General practice-based nurses could play an important role in this educational effort and in informing patients and their caregivers about ACP, which could reduce the workload of GPs. Policymakers and healthcare administrators should support these efforts by providing educational materials and user-friendly IT platforms to improve care continuity and coordination.

The identified risk factors could be used to develop tools to predict unplanned hospital admissions in older adults, allowing targeted interventions. Further research is needed to develop and test interventions aimed at patient education or the provision of person-centred care [25,26]. Moreover, ways to improve collaboration and informational continuity between primary and secondary care and to assess the impact of such initiatives on unplanned hospitalisations should be further explored. Finally, to develop best practice guidelines for continuity and coordination of care, more knowledge is needed from the perspective of older adults at high risk of hospitalisation to detail their ability and motivation to engage in preventive services.

## Conclusion

Unplanned hospital admissions in older adults were perceived to be influenced by patient, healthcare professional, and healthcare organisation-related factors. These factors can help identify older adults at risk. To reduce these admissions, strategies such as ensuring broad access to patients’ clinical information and treatment wishes, improving personal continuity of care, and structural provision of advance care could be beneficial.

## Supplementary Material

Supplemental Material
