# Supplementary material for: What affects unplanned hospital admissions in older adults according to primary healthcare professionals? A focus group study
Source: Eur J Gen Pract. 2026 May 7;32(1):2650928. doi: 10.1080/13814788.2026.2650928 (PMC13159591; doi:10.1080/13814788.2026.2650928)
Supplement: Supplemental Material [file IGEN_A_2650928_SM2092.zip › IGEN_A_2650928_suppl_data/ejgp-2025-0006-File003.docx]

# Appendix 1

## Topic guide

Key question: What are characteristics of older adults with a high risk of unplanned hospital admission?

After introduction of the case history of one of the participants:

- Was this admission expected or not, and why?
- Was this admission preventable? How?
- What admissions do you assume to be preventable?

General questions:

- Which factors are important in estimating the risk of an unplanned admission?
  - Can they be divided into categories?
- Which factor/category has the most influence on unplanned hospital admission?
- Which factors are modifiable? Which are avoidable?
- What could have prevented this admission?
- Can you recall a situation where you were able to prevent an unplanned admission?
  - What did you do?
- What characteristics of you as a professional might influence the risk of unplanned admissions? What could you do about that?
- Which factors could be protective?

For GPs/practice nurses:

- How could the GP electronic health record help with identifying older adults at risk for unplanned admissions?
- Do you envisage this? Would it be helpful? Or rather irritating?
