# Supplementary figures and images for: What affects unplanned hospital admissions in older adults according to primary healthcare professionals? A focus group study
Source: Eur J Gen Pract. 2026 May 7;32(1):2650928. doi: 10.1080/13814788.2026.2650928 (PMC13159591; doi:10.1080/13814788.2026.2650928)

Supplementary File S2 - Coding scheme

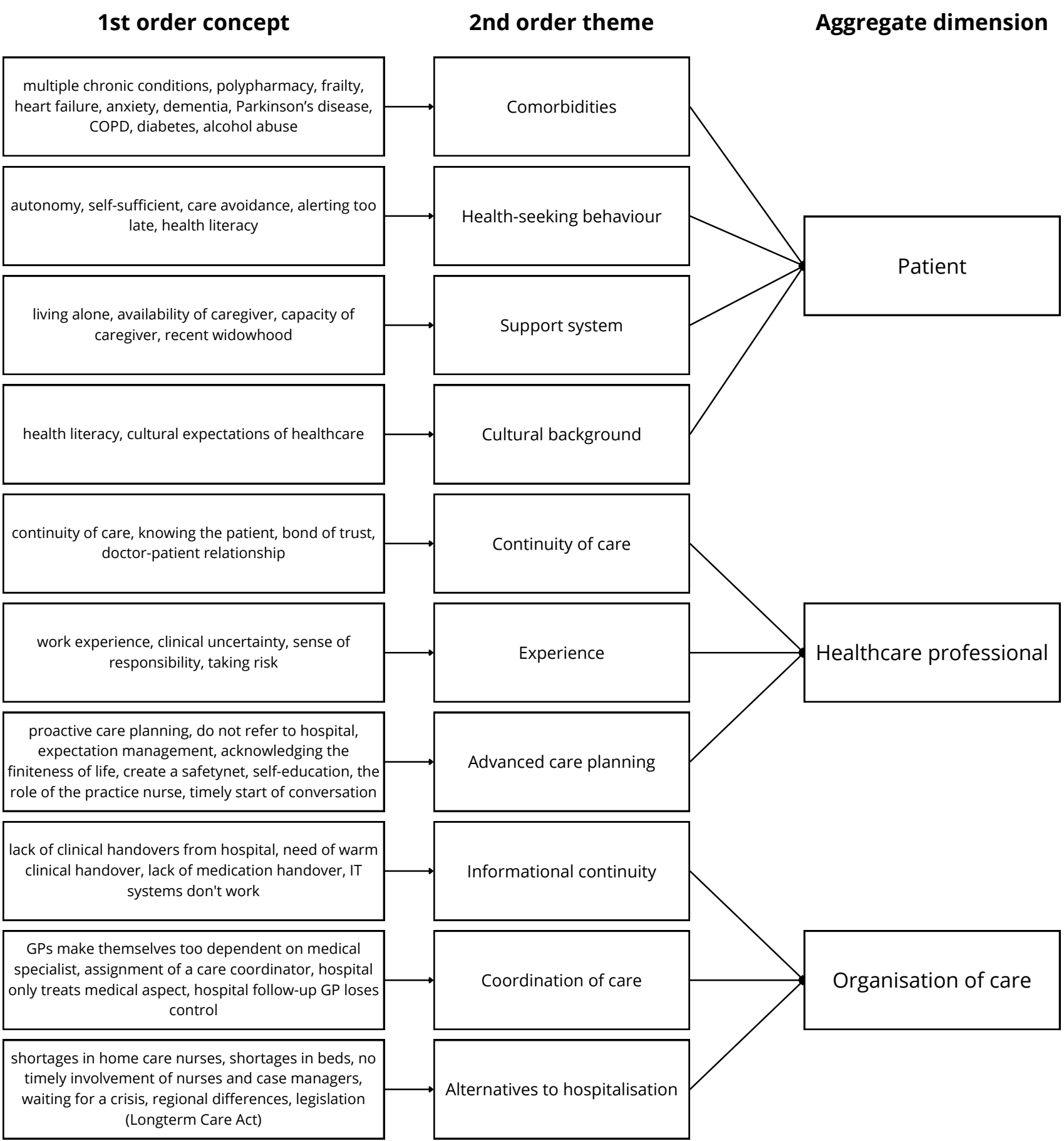

Supplement: Supplemental Material [file IGEN_A_2650928_SM2092.zip › IGEN_A_2650928_suppl_data/ejgp-2025-0006-File004.pdf]
